# {*N*,*N*′-Bis[(*E*)-3-phenyl­allyl­idene]ethane-1,2-diamine}dichloridozinc(II)

**DOI:** 10.1107/S1600536808037860

**Published:** 2008-11-22

**Authors:** Hong-Lan Cai, Bing Liu, Zhi-Dong Lin

**Affiliations:** a School of Chemistry and Materials Science, Ludong University, Shandong 264025, People’s Republic of China; b School of Materials Science and Engineering, Wuhan Institute of Technology, Wuhan 430073, People’s Republic of China

## Abstract

In the title compound, [ZnCl_2_(C_20_H_20_N_2_)], the Zn^II^ atom is four coordinated in a distorted tetra­hedral geometry by two N atoms of the Schiff base ligand and by two Cl atoms. Edge-to-face C—H⋯π inter­actions exist between mol­ecules, with a dihedral angle of 37.8 (1)° between the benzene ring planes and a shortest H⋯centroid distance of 3.62 (5) Å.

## Related literature

For related literature on transition metal complexes of Schiff base ligands, see: Bhatia *et al.* (1981 ▶); Costamagna *et al.* (1992 ▶). For related complexes of ZnCl_2_ with bidentate ligands, see: Tolman *et al.* (1991 ▶); Wang *et al.* (2007 ▶).
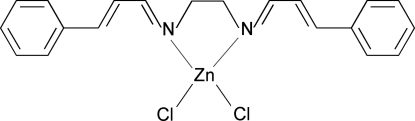


## Experimental

### 

#### Crystal data

[ZnCl_2_(C_20_H_20_N_2_)]
*M* *_r_* = 424.65
Monoclinic, 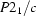

*a* = 7.2140 (8) Å
*b* = 20.265 (2) Å
*c* = 14.0906 (16) Å
β = 94.913 (2)°
*V* = 2052.4 (4) Å^3^
*Z* = 4
Mo *K*α radiation
μ = 1.46 mm^−1^
*T* = 300 (2) K
0.23 × 0.20 × 0.10 mm

#### Data collection

Bruker SMART CCD diffractometer
Absorption correction: multi-scan (*SADABS*; Sheldrick, 1996 ▶) *T*_min_ = 0.730, *T*_max_ = 0.868
15814 measured reflections
4458 independent reflections
3027 reflections with *I* > 2σ(*I*)
*R*_int_ = 0.030

#### Refinement

*R*[*F* ^2^ > 2σ(*F* ^2^)] = 0.047
*wR*(*F* ^2^) = 0.132
*S* = 0.99
4458 reflections
226 parameters
H-atom parameters constrained
Δρ_max_ = 0.50 e Å^−3^
Δρ_min_ = −0.29 e Å^−3^

### 

Data collection: *SMART* (Siemens, 1996 ▶); cell refinement: *SAINT* (Siemens, 1996 ▶); data reduction: *SAINT*; program(s) used to solve structure: *SHELXS97* (Sheldrick, 2008 ▶); program(s) used to refine structure: *SHELXL97* (Sheldrick, 2008 ▶); molecular graphics: *SHELXTL* (Sheldrick, 2008 ▶); software used to prepare material for publication: *SHELXTL*.

## Supplementary Material

Crystal structure: contains datablocks I, global. DOI: 10.1107/S1600536808037860/bi2311sup1.cif

Structure factors: contains datablocks I. DOI: 10.1107/S1600536808037860/bi2311Isup2.hkl

Additional supplementary materials:  crystallographic information; 3D view; checkCIF report

## Figures and Tables

**Fig. 1.**
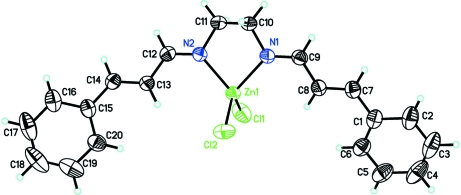
Molecular structure with displacement ellipsoids drawn at 50% probability for non-H atoms.

**Fig. 2.**
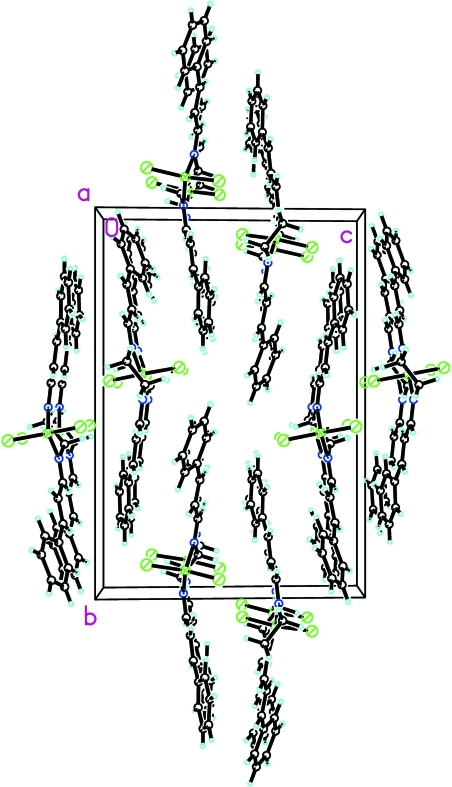
Packing diagram viewed along the a axis.

## supplementary crystallographic information

### Comment

Transition-metal compounds containing Schiff-base ligands play an important role in the development of coordination chemistry related to catalysis and enzymatic reactions, magnetism and molecular architectures (Costamagna *et al.*, 1992; Bhatia *et al.*, 1981). In the title compound (Fig. 1), the Zn^II^ atom is coordinated by a bidentate Schiff-base ligand and two Cl atoms in a slightly distorted tetrahedral geometry. The Zn—Cl bond distances are comparable to those observed in the related compounds [ZnCl_2_(C_12_H_16_BrClN_2_O)] (Wang *et al.*, 2007) and [ZnCl_2_(C_16_H_18_N_4_O)] (Tolman *et al.*, 1991).

### Experimental

Cinnamaldehyde (0.2 mmol, 26.4 mg), ZnCl_2_.6H_2_O (0.1 mmol, 24 mg) and ethylenediamine (0.1 mmol, 6.4 mg) were dissolved in methanol (10 ml). The mixture was stirred for 30 min at room temperature to give a clear yellow solution, which was left in air for a few days to give yellow crystals of the title compound (yield 79%). Elemental analysis calculated: C 56.56, H 4.75, N 6.60%; found: C 56.79, H 4.49, N 6.31%.

### Refinement

H atoms were positioned geometrically, with C—H = 0.93 or 0.97 Å for aromatic and ethyl H, respectively, and constrained to ride on their parent atoms, with *U*_iso_(H) = 1.2*U*_eq_(C).

### Crystal data

**Table:**

[ZnCl_2_(C_20_H_20_N_2_)]	*F*_000_ = 872
*M**_r_* = 424.65	*D*_x_ = 1.374 Mg m^−^^3^
Monoclinic, *P*2_1_/*c*	Melting point: 553 K
Hall symbol: -P 2ybc	Mo *K*α radiation λ = 0.71073 Å
*a* = 7.2140 (8) Å	Cell parameters from 2760 reflections
*b* = 20.265 (2) Å	θ = 2.1–28.1º
*c* = 14.0906 (16) Å	µ = 1.46 mm^−^^1^
β = 94.913 (2)º	*T* = 300 (2) K
*V* = 2052.4 (4) Å^3^	Block, yellow
*Z* = 4	0.23 × 0.20 × 0.10 mm

### Data collection

**Table:**

Bruker SMART CCD diffractometer	4458 independent reflections
Radiation source: fine-focus sealed tube	3027 reflections with *I* > 2σ(*I*)
Monochromator: graphite	*R*_int_ = 0.030
*T* = 300(2) K	θ_max_ = 27.0º
φ and ω scans	θ_min_ = 1.8º
Absorption correction: multi-scanSADABS (Sheldrick, 1996)	*h* = −9→9
*T*_min_ = 0.730, *T*_max_ = 0.868	*k* = −25→24
15814 measured reflections	*l* = −17→12

### Refinement

**Table:**

Refinement on *F*^2^	Secondary atom site location: difference Fourier map
Least-squares matrix: full	Hydrogen site location: inferred from neighbouring sites
*R*[*F*^2^ > 2σ(*F*^2^)] = 0.047	H-atom parameters constrained
*wR*(*F*^2^) = 0.132	*w* = 1/[σ^2^(*F*_o_^2^) + (0.0805*P*)^2^] where *P* = (*F*_o_^2^ + 2*F*_c_^2^)/3
*S* = 0.99	(Δ/σ)_max_ = 0.001
4458 reflections	Δρ_max_ = 0.50 e Å^−^^3^
226 parameters	Δρ_min_ = −0.29 e Å^−^^3^
Primary atom site location: structure-invariant direct methods	Extinction correction: none

### Special details

**Table:**

Geometry. All e.s.d.'s (except the e.s.d. in the dihedral angle between two l.s. planes) are estimated using the full covariance matrix. The cell e.s.d.'s are taken into account individually in the estimation of e.s.d.'s in distances, angles and torsion angles; correlations between e.s.d.'s in cell parameters are only used when they are defined by crystal symmetry. An approximate (isotropic) treatment of cell e.s.d.'s is used for estimating e.s.d.'s involving l.s. planes.
Refinement. Refinement of *F*^2^ against ALL reflections. The weighted *R*-factor *wR* and goodness of fit *S* are based on *F*^2^, conventional *R*-factors *R* are based on *F*, with *F* set to zero for negative *F*^2^. The threshold expression of *F*^2^ > σ(*F*^2^) is used only for calculating *R*-factors(gt) *etc*. and is not relevant to the choice of reflections for refinement. *R*-factors based on *F*^2^ are statistically about twice as large as those based on *F*, and *R*- factors based on ALL data will be even larger.

### Fractional atomic coordinates and isotropic or equivalent isotropic displacement parameters (Å^2^)

**Table:**

	*x*	*y*	*z*	*U*_iso_*/*U*_eq_	
Zn1	0.09704 (4)	0.575283 (15)	0.83248 (3)	0.06115 (16)	
C1	0.2380 (5)	0.84830 (15)	0.8767 (2)	0.0731 (8)	
C2	0.2309 (7)	0.91286 (17)	0.9108 (3)	0.0965 (13)	
H2	0.1260	0.9274	0.9387	0.116*	
C3	0.3789 (9)	0.9553 (2)	0.9034 (4)	0.1214 (18)	
H3	0.3736	0.9982	0.9264	0.146*	
C4	0.5311 (10)	0.9345 (3)	0.8629 (4)	0.1234 (19)	
H4	0.6303	0.9633	0.8585	0.148*	
C5	0.5423 (6)	0.8711 (2)	0.8278 (3)	0.1013 (12)	
H5	0.6482	0.8574	0.8000	0.122*	
C6	0.3962 (5)	0.82863 (17)	0.8343 (2)	0.0822 (9)	
H6	0.4029	0.7861	0.8100	0.099*	
C7	0.0772 (4)	0.80522 (15)	0.8855 (2)	0.0706 (8)	
H7	−0.0286	0.8250	0.9060	0.085*	
C8	0.0665 (4)	0.74079 (14)	0.8672 (2)	0.0674 (8)	
H8	0.1705	0.7195	0.8471	0.081*	
C9	−0.0972 (4)	0.70302 (14)	0.8772 (2)	0.0637 (7)	
H9	−0.2026	0.7248	0.8947	0.076*	
C10	−0.2802 (4)	0.60569 (14)	0.8766 (2)	0.0694 (8)	
H10A	−0.3857	0.6348	0.8626	0.083*	
H10B	−0.2803	0.5912	0.9422	0.083*	
C11	−0.2957 (4)	0.54701 (14)	0.8112 (2)	0.0673 (8)	
H11A	−0.3980	0.5190	0.8267	0.081*	
H11B	−0.3194	0.5615	0.7457	0.081*	
C12	−0.1214 (4)	0.44778 (15)	0.8336 (2)	0.0630 (7)	
H12	−0.2364	0.4268	0.8300	0.076*	
C13	0.0416 (4)	0.40785 (14)	0.8503 (2)	0.0624 (7)	
H13	0.1578	0.4280	0.8537	0.075*	
C14	0.0320 (4)	0.34316 (14)	0.8611 (2)	0.0661 (7)	
H14	−0.0874	0.3255	0.8544	0.079*	
C15	0.1802 (5)	0.29590 (14)	0.8818 (2)	0.0685 (8)	
C16	0.1377 (6)	0.22894 (16)	0.8800 (2)	0.0884 (10)	
H16	0.0154	0.2152	0.8660	0.106*	
C17	0.2773 (10)	0.1826 (2)	0.8991 (3)	0.1219 (18)	
H17	0.2477	0.1380	0.8977	0.146*	
C18	0.4567 (9)	0.2015 (3)	0.9198 (3)	0.131 (2)	
H18	0.5496	0.1700	0.9307	0.158*	
C19	0.5004 (6)	0.2675 (3)	0.9245 (3)	0.1094 (14)	
H19	0.6228	0.2803	0.9405	0.131*	
C20	0.3652 (5)	0.31469 (18)	0.9059 (2)	0.0784 (9)	
H20	0.3967	0.3592	0.9093	0.094*	
Cl1	0.20858 (16)	0.59354 (5)	0.69366 (8)	0.1081 (4)	
Cl2	0.30022 (11)	0.55861 (5)	0.95630 (7)	0.0897 (3)	
N1	−0.1064 (3)	0.64122 (11)	0.86341 (17)	0.0614 (6)	
N2	−0.1194 (3)	0.51002 (10)	0.82321 (16)	0.0593 (6)	

### Atomic displacement parameters (Å^2^)

**Table:**

	*U*^11^	*U*^22^	*U*^33^	*U*^12^	*U*^13^	*U*^23^
Zn1	0.0504 (2)	0.0597 (2)	0.0751 (3)	0.00295 (13)	0.01574 (16)	0.00885 (15)
C1	0.096 (2)	0.0645 (18)	0.0562 (18)	−0.0083 (16)	−0.0095 (16)	0.0046 (14)
C2	0.141 (4)	0.070 (2)	0.074 (3)	−0.011 (2)	−0.012 (2)	−0.0016 (17)
C3	0.186 (6)	0.079 (3)	0.091 (3)	−0.042 (3)	−0.033 (3)	0.002 (2)
C4	0.158 (5)	0.115 (4)	0.088 (3)	−0.066 (3)	−0.040 (3)	0.030 (3)
C5	0.104 (3)	0.116 (3)	0.081 (3)	−0.037 (2)	−0.015 (2)	0.021 (2)
C6	0.088 (2)	0.084 (2)	0.073 (2)	−0.0153 (19)	−0.0024 (18)	0.0062 (18)
C7	0.079 (2)	0.0661 (18)	0.066 (2)	0.0042 (15)	0.0032 (15)	−0.0019 (15)
C8	0.0682 (18)	0.0607 (18)	0.074 (2)	0.0033 (13)	0.0079 (15)	0.0016 (15)
C9	0.0599 (17)	0.0656 (18)	0.066 (2)	0.0055 (13)	0.0109 (14)	0.0036 (14)
C10	0.0510 (16)	0.0672 (17)	0.093 (2)	−0.0010 (13)	0.0202 (15)	−0.0057 (16)
C11	0.0497 (15)	0.0717 (18)	0.080 (2)	0.0018 (13)	0.0009 (14)	0.0018 (16)
C12	0.0601 (17)	0.0664 (17)	0.0626 (19)	−0.0061 (13)	0.0053 (14)	−0.0033 (14)
C13	0.0569 (16)	0.0596 (16)	0.070 (2)	−0.0013 (12)	0.0027 (14)	−0.0044 (14)
C14	0.0681 (18)	0.0637 (18)	0.0661 (19)	−0.0057 (14)	0.0036 (14)	−0.0026 (14)
C15	0.089 (2)	0.0611 (18)	0.0550 (18)	0.0098 (15)	0.0066 (16)	−0.0031 (14)
C16	0.132 (3)	0.067 (2)	0.064 (2)	0.006 (2)	−0.010 (2)	−0.0051 (16)
C17	0.213 (6)	0.073 (2)	0.075 (3)	0.047 (3)	−0.016 (3)	−0.0095 (19)
C18	0.164 (5)	0.140 (4)	0.088 (3)	0.091 (4)	−0.003 (3)	−0.008 (3)
C19	0.093 (3)	0.159 (4)	0.076 (3)	0.046 (3)	0.006 (2)	0.003 (3)
C20	0.075 (2)	0.091 (2)	0.070 (2)	0.0096 (18)	0.0065 (16)	0.0062 (17)
Cl1	0.1177 (8)	0.1124 (7)	0.1025 (8)	0.0353 (6)	0.0584 (6)	0.0377 (6)
Cl2	0.0556 (5)	0.1233 (7)	0.0894 (6)	−0.0061 (4)	0.0012 (4)	0.0130 (5)
N1	0.0555 (13)	0.0600 (14)	0.0702 (16)	0.0012 (10)	0.0137 (11)	0.0006 (11)
N2	0.0549 (13)	0.0592 (14)	0.0641 (15)	0.0023 (10)	0.0068 (11)	0.0023 (11)

### Geometric parameters (Å, °)

**Table:**

Zn1—N2	2.042 (2)	C10—H10A	0.970
Zn1—N1	2.059 (2)	C10—H10B	0.970
Zn1—Cl2	2.2064 (10)	C11—N2	1.473 (3)
Zn1—Cl1	2.2092 (10)	C11—H11A	0.970
C1—C6	1.391 (4)	C11—H11B	0.970
C1—C2	1.396 (5)	C12—N2	1.270 (3)
C1—C7	1.465 (4)	C12—C13	1.431 (4)
C2—C3	1.382 (6)	C12—H12	0.930
C2—H2	0.930	C13—C14	1.322 (4)
C3—C4	1.348 (7)	C13—H13	0.930
C3—H3	0.930	C14—C15	1.447 (4)
C4—C5	1.381 (7)	C14—H14	0.930
C4—H4	0.930	C15—C16	1.391 (4)
C5—C6	1.370 (5)	C15—C20	1.402 (4)
C5—H5	0.930	C16—C17	1.386 (6)
C6—H6	0.930	C16—H16	0.930
C7—C8	1.332 (4)	C17—C18	1.357 (7)
C7—H7	0.930	C17—H17	0.930
C8—C9	1.424 (4)	C18—C19	1.373 (7)
C8—H8	0.930	C18—H18	0.930
C9—N1	1.268 (3)	C19—C20	1.375 (5)
C9—H9	0.930	C19—H19	0.930
C10—N1	1.471 (3)	C20—H20	0.930
C10—C11	1.503 (4)		
			
N2—Zn1—N1	83.04 (9)	N2—C11—C10	108.3 (2)
N2—Zn1—Cl2	113.83 (7)	N2—C11—H11A	110.0
N1—Zn1—Cl2	111.68 (7)	C10—C11—H11A	110.0
N2—Zn1—Cl1	112.73 (8)	N2—C11—H11B	110.0
N1—Zn1—Cl1	113.52 (7)	C10—C11—H11B	110.0
Cl2—Zn1—Cl1	117.26 (4)	H11A—C11—H11B	108.4
C6—C1—C2	118.2 (4)	N2—C12—C13	124.3 (3)
C6—C1—C7	123.4 (3)	N2—C12—H12	117.8
C2—C1—C7	118.5 (4)	C13—C12—H12	117.8
C3—C2—C1	120.4 (5)	C14—C13—C12	121.9 (3)
C3—C2—H2	119.8	C14—C13—H13	119.0
C1—C2—H2	119.8	C12—C13—H13	119.0
C4—C3—C2	120.0 (5)	C13—C14—C15	129.4 (3)
C4—C3—H3	120.0	C13—C14—H14	115.3
C2—C3—H3	120.0	C15—C14—H14	115.3
C3—C4—C5	121.2 (5)	C16—C15—C20	118.2 (3)
C3—C4—H4	119.4	C16—C15—C14	119.0 (3)
C5—C4—H4	119.4	C20—C15—C14	122.8 (3)
C6—C5—C4	119.4 (5)	C17—C16—C15	120.1 (4)
C6—C5—H5	120.3	C17—C16—H16	119.9
C4—C5—H5	120.3	C15—C16—H16	119.9
C5—C6—C1	120.9 (4)	C18—C17—C16	121.0 (4)
C5—C6—H6	119.6	C18—C17—H17	119.5
C1—C6—H6	119.6	C16—C17—H17	119.5
C8—C7—C1	126.9 (3)	C17—C18—C19	119.7 (4)
C8—C7—H7	116.5	C17—C18—H18	120.1
C1—C7—H7	116.5	C19—C18—H18	120.1
C7—C8—C9	122.9 (3)	C18—C19—C20	120.8 (4)
C7—C8—H8	118.6	C18—C19—H19	119.6
C9—C8—H8	118.6	C20—C19—H19	119.6
N1—C9—C8	123.3 (3)	C19—C20—C15	120.1 (4)
N1—C9—H9	118.4	C19—C20—H20	119.9
C8—C9—H9	118.4	C15—C20—H20	119.9
N1—C10—C11	109.2 (2)	C9—N1—C10	119.9 (2)
N1—C10—H10A	109.8	C9—N1—Zn1	130.1 (2)
C11—C10—H10A	109.8	C10—N1—Zn1	109.86 (16)
N1—C10—H10B	109.8	C12—N2—C11	120.0 (2)
C11—C10—H10B	109.8	C12—N2—Zn1	130.7 (2)
H10A—C10—H10B	108.3	C11—N2—Zn1	109.00 (16)
			
C6—C1—C2—C3	−0.9 (5)	C16—C15—C20—C19	1.8 (5)
C7—C1—C2—C3	−179.6 (3)	C14—C15—C20—C19	−179.8 (3)
C1—C2—C3—C4	0.2 (7)	C8—C9—N1—C10	179.1 (3)
C2—C3—C4—C5	0.3 (7)	C8—C9—N1—Zn1	4.1 (4)
C3—C4—C5—C6	0.0 (7)	C11—C10—N1—C9	150.3 (3)
C4—C5—C6—C1	−0.8 (5)	C11—C10—N1—Zn1	−33.8 (3)
C2—C1—C6—C5	1.2 (5)	N2—Zn1—N1—C9	−176.0 (3)
C7—C1—C6—C5	179.8 (3)	Cl2—Zn1—N1—C9	71.1 (3)
C6—C1—C7—C8	9.5 (5)	Cl1—Zn1—N1—C9	−64.2 (3)
C2—C1—C7—C8	−171.8 (3)	N2—Zn1—N1—C10	8.69 (19)
C1—C7—C8—C9	−179.4 (3)	Cl2—Zn1—N1—C10	−104.23 (19)
C7—C8—C9—N1	−177.3 (3)	Cl1—Zn1—N1—C10	120.48 (19)
N1—C10—C11—N2	50.0 (3)	C13—C12—N2—C11	−176.8 (3)
N2—C12—C13—C14	179.6 (3)	C13—C12—N2—Zn1	−3.6 (4)
C12—C13—C14—C15	−177.5 (3)	C10—C11—N2—C12	132.9 (3)
C13—C14—C15—C16	−173.3 (3)	C10—C11—N2—Zn1	−41.7 (3)
C13—C14—C15—C20	8.3 (5)	N1—Zn1—N2—C12	−155.4 (3)
C20—C15—C16—C17	−1.8 (5)	Cl2—Zn1—N2—C12	−44.7 (3)
C14—C15—C16—C17	179.7 (3)	Cl1—Zn1—N2—C12	92.0 (3)
C15—C16—C17—C18	0.1 (6)	N1—Zn1—N2—C11	18.43 (19)
C16—C17—C18—C19	1.8 (7)	Cl2—Zn1—N2—C11	129.09 (17)
C17—C18—C19—C20	−1.9 (7)	Cl1—Zn1—N2—C11	−94.19 (18)
C18—C19—C20—C15	0.0 (6)		
